# State Medicine

**Published:** 1884-04

**Authors:** 


### Editorial.

### STATE MEDICINE.

The phrase "State Medicine" which recently has come into very general use is comparatively new; the idea to which it gives expression is as old as the science of jurisprudence, and is based upon the assumption that the sovereign power in a State or community should take cognizance of the health interests of the people; that it is its duty diligently to inquire into and determine, and within the scope of its authority, to command and enforce, all the procedures necessary to the cure and prevention of disease.

Edicts upon this subject are as old as the laws of Moses, and doubtless constituted a part of "the learning of the Egyptians," with which he was perfectly familiar, and traces of them are to be found in the codes of all nations, proving the universal acquiescence in the assumption, on which has rested all Hygienic legislation; but in no code of laws however recent, does there exist any well-digested system commensurate with the importance of the subject, or with professional opinion of existing necessities.

Two prominent reasons for this imperfection, at least in our own country are apparent. First, exact knowledge of what regulations should be made, and secondly the difficulty of enforcing them.

It is not to be expected that our law-makers possess such exact knowledge of the causes, cure and prevention of disease, as will enable them to enact wise and salutary regulations in regard to them, or that public opinion, which is supposed to be reflected in their legislation, will ever be so enlightened as to sustain and enforce them. In the absence of technical information in the people and their representatives, all efforts at progress must be guided by the opinions of the class of persons whom both will recognize as presumably possessing the required knowledge. From the medical profession alone can such experts be furnished. The great body of doctors are too constantly occupied with the exacting duties of their calling to give the requisite time and attention to this special subject to make them all safe guides in any emergency. An accidental discovery or chance hit in practice may result in valuable contributions to curative and preventive medicine, but the conclusive and exact knowledge upon which stable legislation can be predicated must be supplied by comparatively few, whose opportunities and tastes have led to the investigation of the causes and nature of disease with philosophical impartiality and from the highest standpoint of observation, and whose reputation for wisdom and exact truth is so well established as to command popular legislative and professional approval.

The statutes of the United States and of each State of the American Union demonstrate that the law-makers are not unwilling to adopt any well considered suggestion promotive of the public health; indeed, they have often exhibited too great anxiety by the adoption of many impracticable and visionary schemes resulting in no other good than as the lesson of a useless experiment.

Without specifying particular enactments in different States, it may be stated that the principle of American law asserts the authority of the sovereignty to enforce all measures necessary to limit the proportions of preventable disease. It subjects all states of property and all such modes of personal action or inaction as may endanger public health to summary procedure. It gives plenary powers to local authorities and imposes upon them the obligation to suppress all nuisances and to provide such establishments as the public health requires, and for such purposes it limits the private rights and control of property and the freedom of persons. It interferes in the interest of health between parent and child, between employer and employé, between vendor and purchaser. It restricts the sale of poisons and makes penal the sale or offer for sale of adulterated food or drink.

This summary shows that beyond doubt legislators regard the health of the people as a paramount interest, national and personal, and desire to guard it with all practicable securities against trespasses, casualties, neglects and frauds.

Medical science has not yet attained the necessary precision and exactness in all instances to guide their action and respond to their benevolent intentions. The methods of preventing, of absolutely eradicating, a few diseases are well known, as well as the means of lessening the prevalence and mortality of others, but a vast region of the dark unknown lies before medical scientists, to be explored and illuminated, before they can pretend to stand between the living and the dead, and, clothed with the power of the law, prevent the progress of disease. Much has been accomplished in this direction, probably more of exact knowledge gained in the last thirty years than in all preceding time, but "the harvest truly is plenteous and the laborers are few." Unaided individual effort cannot soon accomplish so vast a task. Whatever it can do the past gives assurance will be faithfully attempted, but in view of the magnitude of the interests involved, it would appear that the aid of the State should be invoked to hasten the realization of hopes so fondly and reasonably cherished.

The history of legislative action on this subject leaves but little doubt that the *single* or *respective* sovereignties of our country, upon reasonable assurance of its propriety, would not hesitate to employ at honorable compensation a sufficient number of competent experts to investigate the causes of all disease and by intelligently conducted experiments arrive at certainty as to the best means of prevention, and thus by demonstration satisfy the legislative and popular mind.

The establishment of Boards of Health by the United States Government and by many of the individual States, shows the activity of the public thought in this direction. Some of them have lost, to some extent, the confidence of the people, from the too extravagant and impatient expectation of immediate beneficial results. They have only recently begun to form a true estimate of the inestimable benefits which preventive medicine can possibly afford them, and supposed that medical boards had only to formulate edicts in accordance with certain well known principles; their disappointment is not wonderful when they learned that many of these principles are still in doubt, that all existing *true knowledge* of morbic causes has come by slow degrees and as a part of the development by which science has little by little, during the ages, been building itself up, and that the primary purpose of these boards is to concentrate the rays of the highest human intelligence upon the brightest focus of human hope, and to hasten by centuries the inauguration of the time when all deleterious physical agencies shall be shorn of their power to harm.

With such corps of experts wisely organized and fitly maintained, the public mind would soon become instructed, confidence would follow instruction, and all things which science thus voiced would be made the behests of law.

In republics, the people not only make the law, but are expected to enforce it; its requirements therefore must be backed up by overwhelming popular approbation. There is probably no State which does not furnish examples of this necessity; instances in which laws have remained for years upon the statute books without attention or enforcement. Acts in relation to legal medicine above all others require this support. They interfere more with individual freedom, conflict oftener with social habits and prejudices, and oftener disturb the inertia of every-day private life than any other branch of legislation. New laws, like unaccustomed taxes, are odious, and are alone justified by high necessity, and for their observance are dependent upon popular information and conviction of their utility. The opinion of eminent scientists, and results of their experiments widely published, would sooner or later carry with it professional and general acquiescence.

This obstacle to the progress of sanitary legislation may be illustrated by referring to two examples. The means of preventing small-pox, and of absolutely annihilating that disease, are as well known as any scientific fact can possibly be, but after a century of delay, in no State of the Union is vaccination made compulsory. Nothing short of this gives to society its full protective power.

Personal freedom is a right so sacred that authority hesitates to invade it. Even professional opinion is not agreed as to the proper manner of performing vaccination, and many still shrink from outraging maternal affection by making more than a single puncture. In other countries legislation has been bolder; and upon the recommendation of acknowledged experts, it can scarcely be doubted that our own might gain courage enough to encounter the frowns and tears of mothers and the possible screams of their infants.

But in the other example is required a sublimity of valor not to be hoped for, at least in a Georgia legislator, and a degree of intelligence and devotion to the public good rarely to be found in a Georgia "nigger."

Though somewhat infrequent, the most appalling of all diseases is Hydrophobia; its cause is well known, the methods of preventing it address themselves to the common sense of everybody, the experience of other countries, Holland especially, demonstrates the practicability of its perfect extermination. A public law which would propose to protect society against its ravages would encounter most formidable opposition, and an attempt to enforce it would endanger rebellion; men, children, negroes and dogs would resist it. The opposition of all the three first might be braved, but any infringement upon the prescriptive rights and privileges of the last has been always hitherto successfully resisted.

Regulations intended to prevent other diseases more prevalent but whose causes are less known, would necessarily conflict to a greater extent with social customs and popular prejudice, and become prudent and practicable only on the recommendation of the highest scientific authority; which will ultimately carry with it the assent and support of the public.

Without criticising the action of medical boards anywhere, it is possible that the comparative want of success and loss of popularity which has attended their action has been partly caused by their having attempted too much. So far as scientific investigation or popular observation has established absolute truth, energetic action should be prompt and immediate, and the strong support of the law invoked, but in the vast region of the doubtful or the unknown their recommendation should be cautious and tentative, alarming as little as may be the sensitiveness of the people to inquisitorial officiousness, but evincing in the clearest manner intelligent and tireless activity in the philanthropic objects of their creation.

Some mistakes or repeated failures often furnish instructive lessons of future progress, and constitute no good reason for relaxation of effort or despair. No field of scientific investigation is fuller of hope than the so-called science of State Medicine. Aided or unaided by the fostering bounty of Bodies Politic it will be kept abreast with the advance of other departments of useful knowledge, and the suffering and necessities of a common humanity will surely, if slowly, educate the popular intelligence up to the requirements of scientific discovery.

